# A versatile salt-based method to immobilize glycosaminoglycans and create growth factor gradients

**DOI:** 10.1007/s10719-019-09872-4

**Published:** 2019-05-04

**Authors:** Danique J. Hof, Elly M. M. Versteeg, Chris H. A. van de Lest, Willeke F. Daamen, Toin H. van Kuppevelt

**Affiliations:** 1 Department of Biochemistry, Radboud Institute for Molecular Life Sciences, Radboud university medical center, PO Box 9101, 6500 HB Nijmegen, The Netherlands; 2 Present Address: Department of Equine Sciences and Department of Biochemistry and Cell Biology, Faculty of Veterinary Medicine, Utrecht University, Utrecht, The Netherlands

**Keywords:** Glycosaminoglycan, Growth factor gradient, Hofmeister series of salts, Immobilization

## Abstract

Glycosaminoglycans (GAGs) are known to play pivotal roles in physiological processes and pathological conditions. To study interactions of GAGs with proteins, immobilization of GAGs is often required. Current methodologies for immobilization involve modification of GAGs and/or surfaces, which can be time-consuming and may involve specialized equipment. Here, we use an efficient and low-cost method to immobilize GAGs without any (chemical) modification using highly concentrated salt solutions. A number of salts from the Hofmeister series were probed for their capacity to immobilize heparin and chondroitin-6-sulfate on microtiter plates applying single chain antibodies against GAGs for detection (ELISA). From all salts tested, the cosmotropic salt ammonium sulfate was most efficient, especially at high concentrations (80–100% (v/v) saturation). Immobilized GAGs were bioavailable as judged by their binding of FGF2 and VEGF, and by their susceptibility towards GAG lyases (heparinase I, II and III, chondroitinase ABC). Using 80% (v/v) saturated ammonium sulfate, block and continuous gradients of heparin were established and a gradient of FGF2 was created using a heparin block gradient as a template. In conclusion, high concentrations of ammonium sulfate are effective for immobilization of GAGs and for the establishment of gradients of both GAGs and GAG-binding molecules, which enables the study to the biological roles of GAGs.

**Electronic supplementary material:**

The online version of this article (10.1007/s10719-019-09872-4) contains supplementary material, which is available to authorized users.

## Introduction

Glycosaminoglycans (GAGs) are an important class of biomolecules, consisting of repeating disaccharide units. The highly negatively charged GAGs are ubiquitously present on cell surfaces and within the extracellular matrix (ECM), where they interact with a variety of structural and signaling proteins, including collagens, growth factors and chemokines [1]. Through these interactions they play a pivotal role in various physiological processes, like ECM remodeling [2], immune responses [3] and blood clotting [4]. In addition, GAGs are also associated with a number of pathological conditions, e.g. Alzheimer’s disease [5], cancer [6] and chronic obstructive pulmonary disease [7]. Furthermore, GAGs are used as supplements and coatings in (bio)materials to gain specific biological properties improving biocompatibility and supporting tissue repair [2, 8, 9].

The mechanisms underlying the biological roles of GAGs, including their interactions with biomolecules and cells, largely remain to be elucidated. To be able to study these interactions, GAGs need to be immobilized on surfaces, for example on a microtiter plate to perform an enzyme-linked immunosorbent assay (ELISA). Due to their negative charges GAGs poorly bind to apolar surfaces like polystyrene microtiter plates. A variety of GAG immobilization methods have been developed by modifying GAGs themselves [10–14], and/or by modifying the surface to which the GAGs are bound [15–18]. GAGs are generally covalently bound by modification of the reducing end of the polysaccharide [11, 13–16, 19] or via the carboxylic groups [10, 12, 14, 18]. Methods to immobilize GAGs non-covalently include the use of microtiter plates coated with allyl amine applying cold plasma polymerization techniques requiring a high-cost setup or commercially available allyl amine coated plates [17].

We previously reported on the use of high salt solutions for linking negatively charged macrobiomolecules, including glycosaminoglycans, to various surfaces (patents US 6,180,769 B1 and US 6,933,379 B2) [20, 21]. This method does not require additional modification of the GAG nor the surface, as the GAG can be immobilized directly due to the presence of the salt. We here extend the use of the salt-based immobilization method including the preparation of gradients of GAGs and GAG-binding proteins.

## Materials and methods

### Materials

Ammonium sulfate ((NH_4_)_2_SO_4_), ammonium chloride (NH_4_Cl), sodium sulfate (Na_2_SO_4_), magnesium sulfate (MgSO_4_), sodium chloride (NaCl), lithium chloride (LiCl), magnesium chloride (MgCl_2_), magnesium sulfate (MgSO_4_), magnesium acetate (Mg(Ac)_2_), potassium chloride (KCl), guanidinium chloride (GuHCl), calcium acetate (CaAc) and bovine serum fraction V (BSA) were purchased from Merck (Darmstadt, Germany). Calcium sulfate (CaSO_4_), calcium chloride (CaCl_2_), Tween-20® and diethanolamine were provided by Sigma-Aldrich (St. Louis, MO, USA). *p*-Nitrophenyl phosphate disodium salt was obtained from MP Biomedicals (Santa Ana, CA, USA). UltraPure® Tris was received from Invitrogen™ (Thermo Fisher Scientific, Waltham, MA, USA). Heparin sodium was from Organon (Oss, the Netherlands), heparan sulfate (HS) sodium salt from bovine kidney and chondroitin-6-sulfate (CSC) sodium salt from shark cartilage from Sigma-Aldrich and dermatan sulfate (DS) sodium salt from Celsus laboratories, Inc. (Cincinnati, OH, USA). Heparinase I, II and II were provided by IBEX Technologies, Inc. (Montreal, QC, Canada) and chondroitinase ABC by Sigma-Aldrich. Recombinant human VEGF 165 protein was from R&D systems (Minneapolis, MN, USA). Recombinant rat FGF2 was produced in *Escherichia coli* using a FGF2 expression construct cloned in the prokaryotic vector pQE16, which was a gift from the Department of Pathology, Radboud university medical center, Nijmegen, The Netherlands [22]. The single chain variable fragment (scFv) antibodies HS4C3V, IO3H10 and GD3A12 specific for heparin/HS, CS and DS, respectively, were produced as described and contain a VSV tag [23, 24]. The specificity of the scFv antibodies were assessed previously by biochemical/histochemical methods, including ELISA’s and immunohistochemistry [23, 25, 26]. To detect the scFv antibodies, anti-VSV tag IgG from the mouse hybridoma cell line P5D4 from the American Type Culture Collection (Rockville, MD, USA) or polyclonal rabbit anti-VSV-G antibody from Rockland Immunochemicals Inc. (Limerick, PA, USA) were used. Rabbit anti-fibroblast growth factor-basic (1–24) antibody (immunogen is a synthetic peptide sequence corresponding to bovine FGF2 (1–24)), rabbit anti-goat IgG-alkaline phosphatase (AP) conjugated and goat anti-rabbit IgG-AP were obtained from Sigma-Aldrich. Polyclonal goat anti-human VEGF 165 antibody was from R&D systems and polyclonal goat anti-mouse IgG-AP conjugated from Dako, Agilent Pathology Solutions (Santa Clara, CA, USA). Goat anti-mouse IgG conjugated with IRDye 800CW and goat anti-rabbit IgG conjugated with IRDye 680CW were provided by LI-COR Biotechnology ((Westburg b.v.), Leusden, the Netherlands). For all ELISA assays, clear flat bottom 96-wells Microlon® ELISA plates (Item No.: 655092) from Greiner Bio-One B.V. (Alphen aan de Rijn, the Netherlands) were used.

### Optimization of saturated salt solution for GAG immobilization

The optimal saturated salt solution to coat GAG to 96-wells microtiter plates was determined using an indirect ELISA. First, microtiter plates were incubated with various amounts of heparin or CSC (500–0 ng/well) dissolved in 80% (v/v) saturated solutions of various salts i.e. (NH_4_)_2_SO_4,_ NH_4_Cl, Na_2_SO_4_, MgSO_4_, NaCl, LiCl, MgCl_2_, MgSO_4_, KCl, GuHCl, CaSO_4_, CaCl_2_ and MilliQ water. Incubation was overnight on a wet cloth to minimize fringe effects. The plates were rinsed six times with PBS containing 0.1% (v/v) Tween-20® (PBST). After blocking with 1% (w/v) BSA in PBST for 60 min, the scFv antibodies HS4C3V and IO3H10 in 1% (w/v) BSA in PBST were allowed to bind to the coated heparin and CSC respectively for 60 min. Subsequently, the plates were incubated with mouse anti-VSV antibody P5D4 in 1% (w/v) BSA in PBST and anti-mouse IgG-AP in 1% (w/v) BSA in PBST for 60 min. The bound AP conjugated antibody was detected by addition of 100 μl to each well of 1 mg *p*-nitrophenyl phosphate/ml in 1 M diethanolamine with 0.5 mM MgCl_2_, pH 9.8. The conversion of *p*-nitrophenyl phosphate into of *p*-nitrophenol by AP produces a yellow color, which was measured at 405 nm.

The optimal degree of salt saturation (v/v) to immobilize heparin, CSC and HS was determined for (NH_4_)_2_SO_4_ using the same method as described above and compared to NaCl as a less effective salt. The assessed degrees of saturation used were 100% (v/v), 90% (v/v), 80% (v/v), 70% (v/v), 60% (v/v), 50% (v/v) and 25% (v/v).

Each condition was performed in duplicate and the experiment was repeated three times (n = 3) for heparin and CSC and four times (n = 4) for HS. Data were normalized against the absorbance value at 405 nm observed for 500 ng GAGs in 80% (v/v) saturated (NH_4_)_2_SO_4_ solution, which was added to each plate as an internal reference.

### Enzymatic digestion of immobilized GAGs

A 96-wells microtiter plate was incubated with 1 μg/well of heparin, HS, CSC or DS in 80% (v/v) saturated (NH_4_)_2_SO_4_ solution overnight. The plates were rinsed six times with PBST after each incubation. After blocking the plate with 1% (w/v) BSA in PBST for 90 min, the GAG-coated wells were either incubated with a combination of heparinase I, II and III (0.02 IU of each enzyme in 1 ml 100 mM NaAc with 0.2 mM CaCl_2_ pH 7.2), with chondroitinase ABC (0.02 IU in 1 ml 25 mM Tris and 2 mM Mg(Ac)_2_ pH 8), or with digestion buffer only, overnight at 37 °C. Subsequently, the remaining GAGs were detected with the scFv antibodies HS4C3V, IO3H10 and GD3A12 using the indirect ELISA method described above. Each condition was performed in duplicate and the experiment was repeated three times (n = 3).

### Growth factor binding of immobilized GAGs

Microtiter plates were incubated with 500 ng/well of heparin, HS, CSC or DS in 80% (v/v) saturated (NH_4_)_2_SO_4_ overnight. The plates were rinsed six times with PBST after each incubation. After blocking with 1% (w/v) BSA in PBST for 60 min, the GAG-coated microtiter plates coated were incubated with 25 ng/well of either rat FGF2 or human VEGF in 1% (w/v) BSA in PBST for 60 min. The bound growth factors were detected with rabbit anti-fibroblast growth factor-basic (1–24) and goat anti-human VEGF 165 antibody and subsequently rabbit anti-goat and goat anti-rabbit IgG-AP using the indirect ELISA method described above. Each condition was performed in duplicate and the experiment was repeated three times (n = 3).

### Creating a gradient of immobilized heparin and FGF2

A block gradient of heparin was created in a 24-wells Greiner CELLSTAR® cell suspension plate by diluting 2 μg of heparin in 100 μl of 80% (v/v) saturated (NH_4_)_2_SO_4_ each hour with an additional 100 μl of 80% (v/v) saturated (NH_4_)_2_SO_4_. To create a FGF2 gradient, a heparin block gradient was incubated with 400 μl of 200 ng/ml FGF2 in 0.4% (w/v) BSA in PBS for 60 min. The plate was rinsed six times with PBST, blocked with 1% (w/v) BSA in PBST for 60 min and incubated with anti-fibroblast growth factor-basic (1–24) antibody and goat anti rabbit IgG conjugated IRDye 800CW, 60 min each, and rinsing with PBST after each incubation. To create a continuous gradient of heparin, 2 μg of heparin in 100 μl of 80% (v/v) saturated (NH_4_)_2_SO_4_ was placed at one side of a well of a 24-wells cell suspension plate. The heparin was immediately diluted with 80% (v/v) saturated (NH_4_)_2_SO_4_ solution at a rate of 0.1 ml/h using a pump system (New era pump systems inc, model number NE-300). After the whole surface of the well was covered with (NH_4_)_2_SO_4_, the plates were washed six times with PBST and blocked with 1% (w/v) BSA in PBST 60 min. The gradient of heparin was visualized by successive incubations with HS4C3V, P5D4 and goat anti mouse IgG conjugated with IRDye 800CW in 1% (w/v) BSA in PBST for 60 min each and rinsing with PBST after each incubation. The IRDye staining was visualized using the Odyssey CLx imaging system, which was further processed and analyzed with Fiji 1.51n software.

### Creating patterns of immobilized heparin and CSC

A clover-shaped mold was designed with Autodesk® Tinkercad® and 3D printed with polylactic acid using an adapted Anet® A8 3D printer (Shenzhen Anet Technology Co., Ltd., Shenzhen, Guangdong, China) (Fig. 6c). The mold was placed at the bottom of a 24-wells Greiner CELLSTAR® cell suspension plate (Fig. 6d). A 400 μl solution of 20 μg/ml of heparin in 80% (v/v) saturated (NH_4_)_2_SO_4_ was placed inside the mold and incubated overnight. After removing the mold and rinsing six times with PBST, the plate was blocked with 1% BSA in PBST. The pattern of immobilized heparin was visualized using the antibodies HS4C3V, P5D4 and goat anti mouse IgG conjugated with IRDye 800CW in 1% (w/v) BSA in PBST. The reverse pattern was created by performing the same experiment with CSC placed on the outside of the mold in a new well of the 24-wells plate. The reverse clover pattern of CSC was detected with IO3H10, polyclonal rabbit anti-VSV-G antibody and goat anti rabbit IgG conjugated with IRDye 680CW in 1% (w/v) BSA in PBST. The IRDye staining was visualized using the Odyssey CLx imaging system, which was further processed and analyzed with Fiji 1.51n.

## Results

### Immobilization of GAGs

**Fig. 1:**
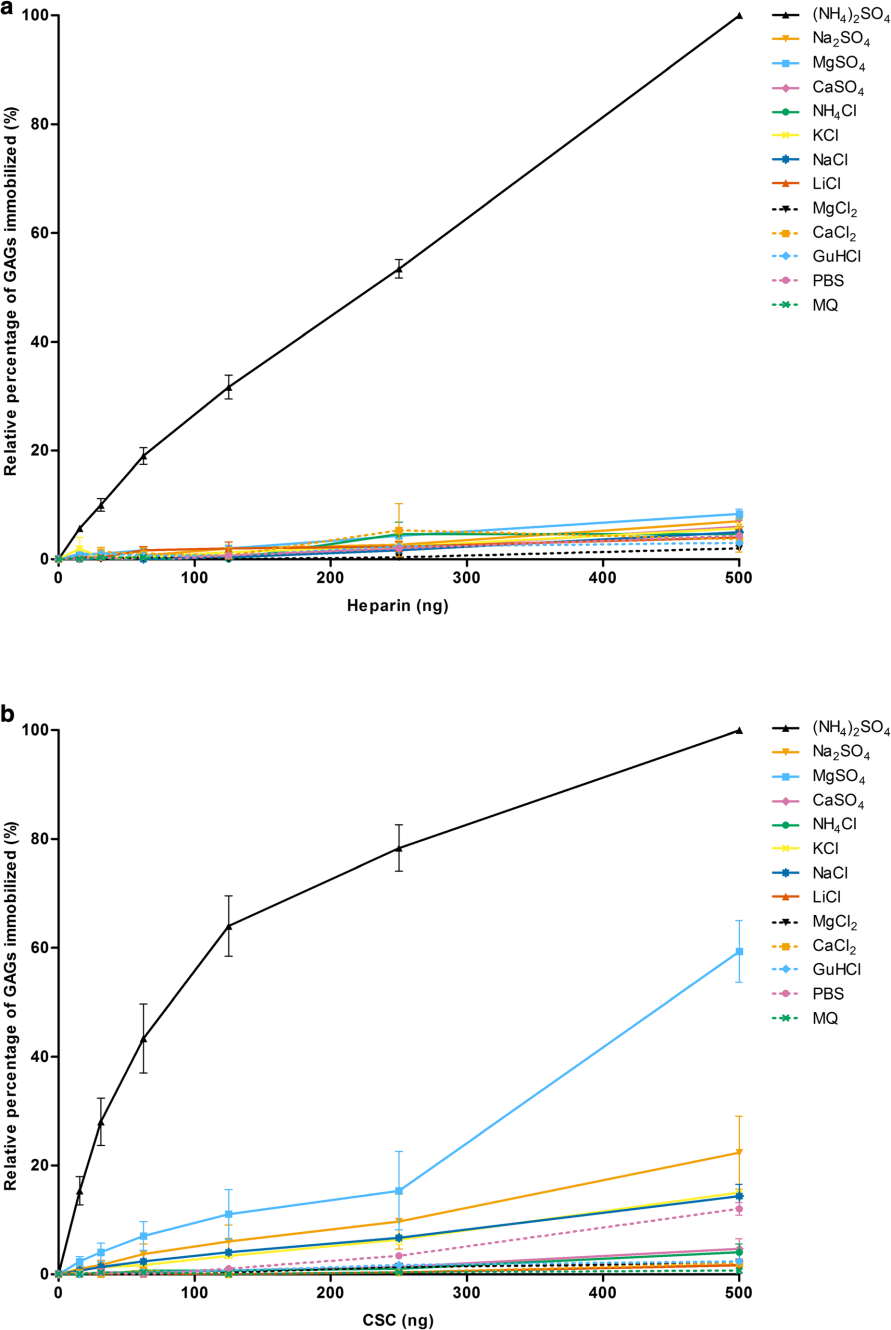
The immobilization of (**a**) heparin and (**b**) chondroitin-6-sulfate (CSC) on 96-wells microtiter plates using various 80% (v/v) saturated salt solutions. Immobilized heparin and CSC (incubated at 0–500 ng/well) were detected via an indirect ELISA using the scFv antibodies HS4C3V and IO3H10, respectively. Data were normalized against the absorbance value at 405 nm observed for 500 ng GAGs in 80% (v/v) saturated (NH_4_)_2_SO_4_ solution, which was added to each plate as an internal reference. Data are presented as mean with standard error of the mean (n = 3 in duplicate). MQ = MilliQ water

Heparin and chondroitin-6-sulfate (CSC) were immobilized on polystyrene plates using 80% (v/v) saturated solutions of various salts applying 0–500 ng GAG/well, and immobilization was assessed by an indirect ELISA assay using single chain antibodies against heparin and CSC (See Materials and methods). In comparison with other salts of the Hofmeister series ammonium sulfate ((NH_4_)_2_SO_4_) was superior in immobilizing heparin and CSC (Fig. 1a, b). The amount of GAGs immobilized by (NH_4_)_2_SO_4_ was dose-dependent. Increasing the concentration of heparin in the coating solution resulted in a near linear increase of immobilized heparin, whereas for CSC an initial steep increase was observed followed by a more gradual increase towards a maximum value. Use of other (cosmotropic) salts resulted in only minor amounts of GAGs immobilized at the given GAG concentration range. Only at the highest amount of CSC (500 ng/well), 80% (v/v) saturated magnesium sulfate (MgSO_4_) gave a positive signal.

**Fig. 2:**
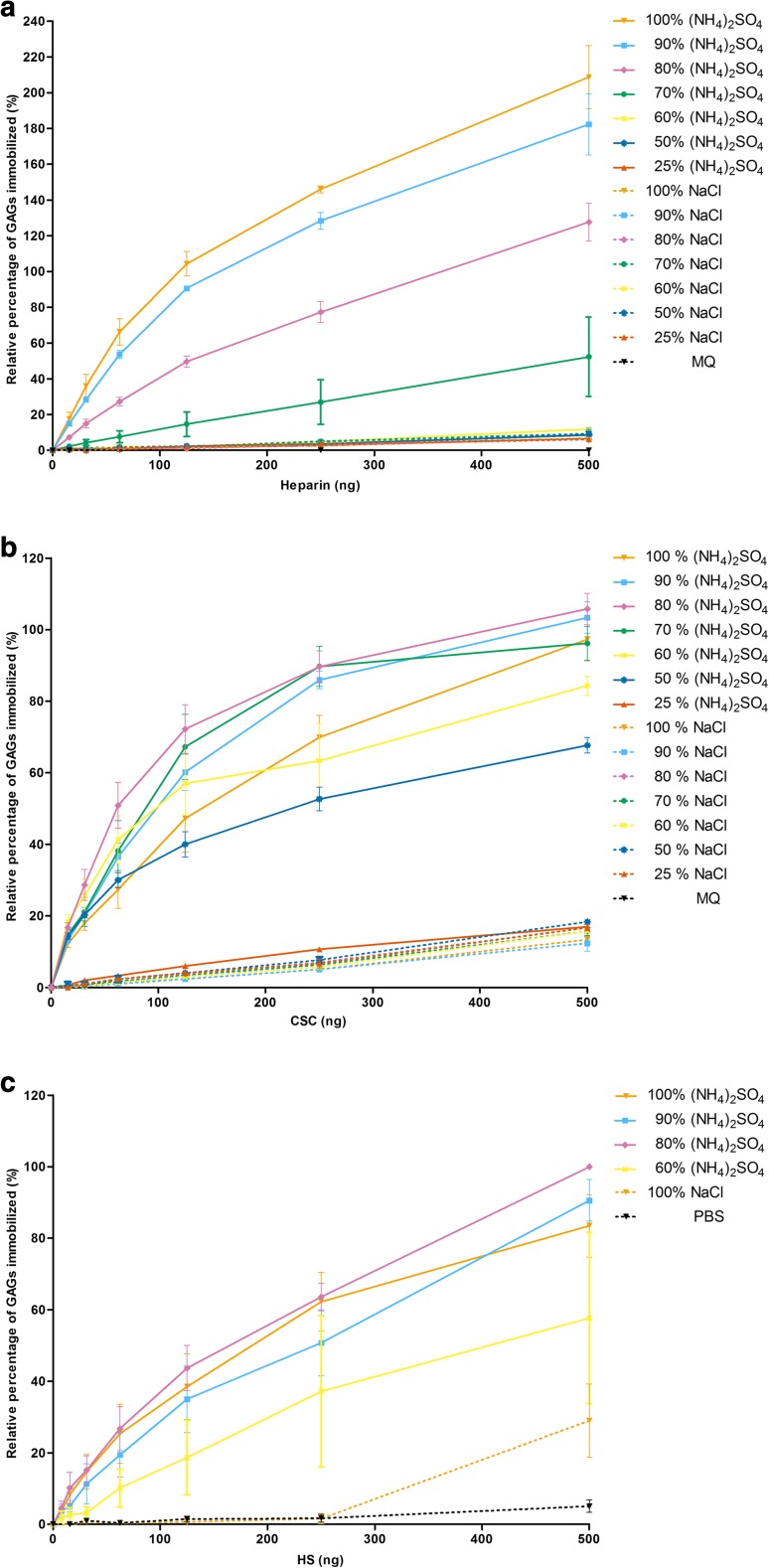
The immobilization of (**a**) heparin, (**b**) chondroitin-6-sulfate (CSC) and (**c**) heparan sulfate (HS) on 96-wells microtiter plates using various saturation percentages (v/v) of (NH_4_)_2_SO_4_ and NaCl solutions. Immobilized glycosaminoglycans (GAGs) were detected via an indirect ELISA using the scFV antibodies HS4C3V for heparin and HS and IO3H10 for CSC. Data were normalized against the absorbance value at 405 nm observed for 500 ng GAGs in 80% (v/v) saturated (NH_4_)_2_SO_4_ solution, which was added to each plate as an internal reference. Data are presented as mean with standard error of the mean (heparin, CSC n = 3 in duplicate; HS n = 4 in duplicate). MQ = MilliQ water

Having identified (NH_4_)_2_SO_4_ as the most efficient salt for the immobilization of GAGs, the optimum salt saturation percentage was assessed. Heparin, CSC and heparan sulfate (HS) were immobilized using various saturation percentages of (NH_4_)_2_SO_4_ and of sodium chloride (NaCl) as a less effective salt. The 100, 90, 80 and 70% (v/v) saturated solutions of (NH_4_)_2_SO_4_ were more efficient in immobilizing heparin compared to solutions of with lesser degrees of saturation and all solutions of NaCl (Fig. 2a). For the immobilization of CSC, solutions of (NH_4_)_2_SO_4_ of 50, 60, 70, 80, 90 and 100% (v/v) saturation were most efficient, where none of the NaCl solutions showed more than 20% of the maximum binding using (NH_4_)_2_SO_4_ (Fig. 2b). The overall highest percentage of immobilization for heparin was obtained using 100% (v/v) (NH_4_)_2_SO_4_, while for CSC and HS (Fig. 2c) this was 80% (v/v). For further experiment we used an 80% (v/v) saturated (NH_4_)_2_SO_4_ solution for immobilization of all GAGs.

### Enzymatic digestion of immobilized GAGs

**Fig. 3:**
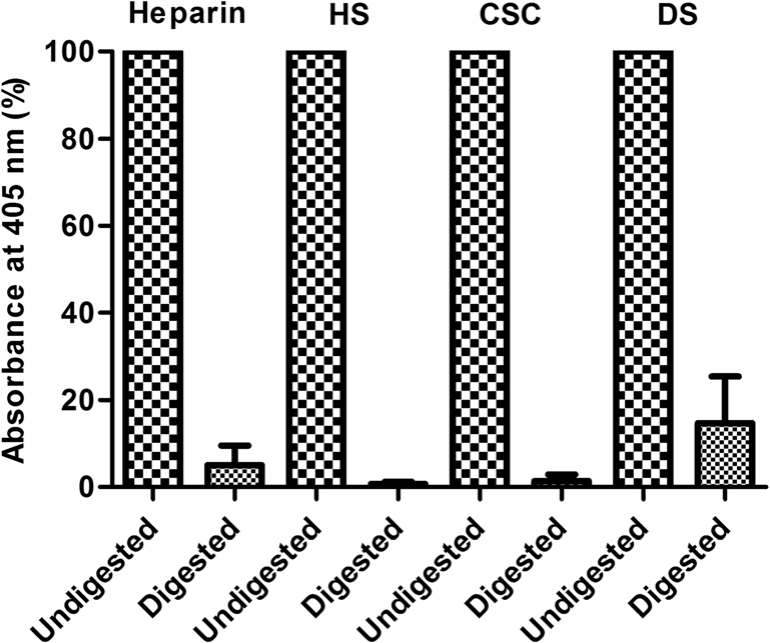
Bioavailability of immobilized glycosaminoglycans. Immobilized heparin and heparan sulfate (HS) (both incubated at 1 μg/well) were digested with heparinase I, II and III. Immobilized chondroitin-6-sulfate (CSC) and dermatan sulfate (DS) were digested with chondroitinase ABC. The undigested and digested GAGs were detected via an indirect ELISA using the scFv antibodies HS4C3V for heparin and HS, IO3H10 for CSC and GD3A12 for DS. Data were normalized against the absorbance values at 405 nm observed for the corresponding non-digested glycosaminoglycan (GAG), and which were set at 100%. Data are given as mean with standard deviation in error bars (n = 3 in duplicate)

To verify whether immobilized GAGs were still bioavailable for glycolytic enzymes, immobilized heparin and HS were incubated with a mix of heparinase I, II and III. Immobilized CSC and dermatan sulfate (DS) were incubated with chondroitinase ABC for this reason. After digestion, a major reduction in immobilized GAGs was observed (Fig. 3), indicating good accessibility for enzymatic degradation.

### Growth factor binding to immobilized GAGs

**Fig. 4:**
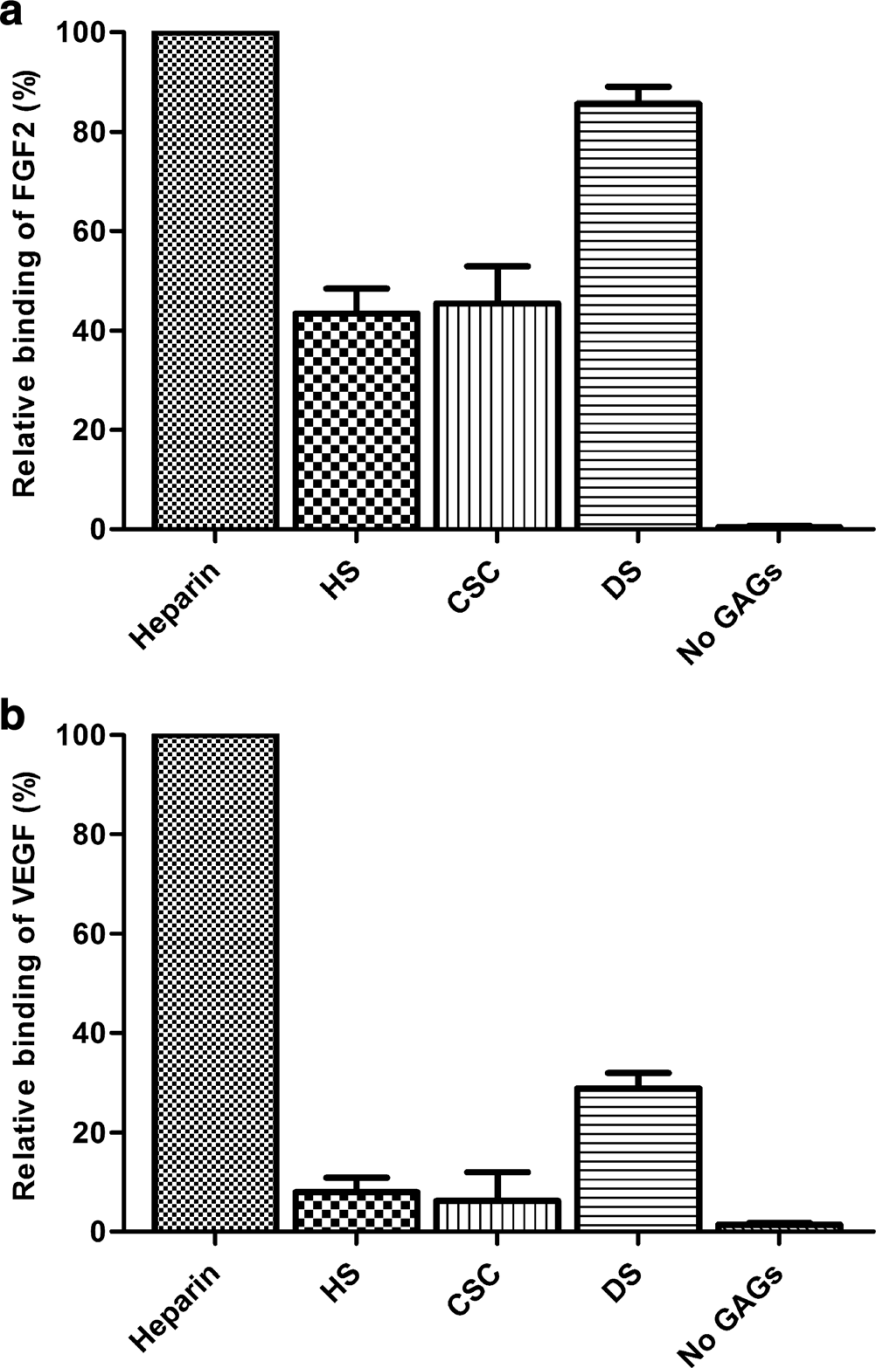
Binding of (**a**) FGF2 and (**b**) VEGF (incubated at 25 ng/well) to immobilized heparin, heparan sulfate (HS), chondroitin-6-sulfate (CSC), dermatan sulfate (DS) (incubated at 500 ng/well) and to the well without glycosaminoglycans (no GAGs). The bound growth factors were detected via an indirect ELISA using rabbit anti-fibroblast growth factor-basic and goat anti-human VEGF 165 antibody. Data were normalized against the maximum absorbance values obtained at 405 nm, which in this case were the growth factors bound to heparin. Data are presented as the relative binding of the growth factors corrected for the blank (coating with 80% (v/v) (NH_4_)_2_SO_4_ without growth factor). Data are given as mean with standard deviation in error bars (n = 3 in duplicate)

To evaluate the capacity of the immobilized GAGs to bind growth factors, FGF2 and VEGF were allowed to bind to immobilized heparin, HS, CSC and DS. All immobilized GAGs were able to bind FGF2 and VEGF, with heparin resulting in the highest amounts of detected growth factors (Fig. 4a, b). Furthermore, a higher amount of FGF2 and VEGF were detected on immobilized DS compared to HS and CSC.

### Creation of a gradient of immobilized heparin and FGF2

**Fig. 5:**
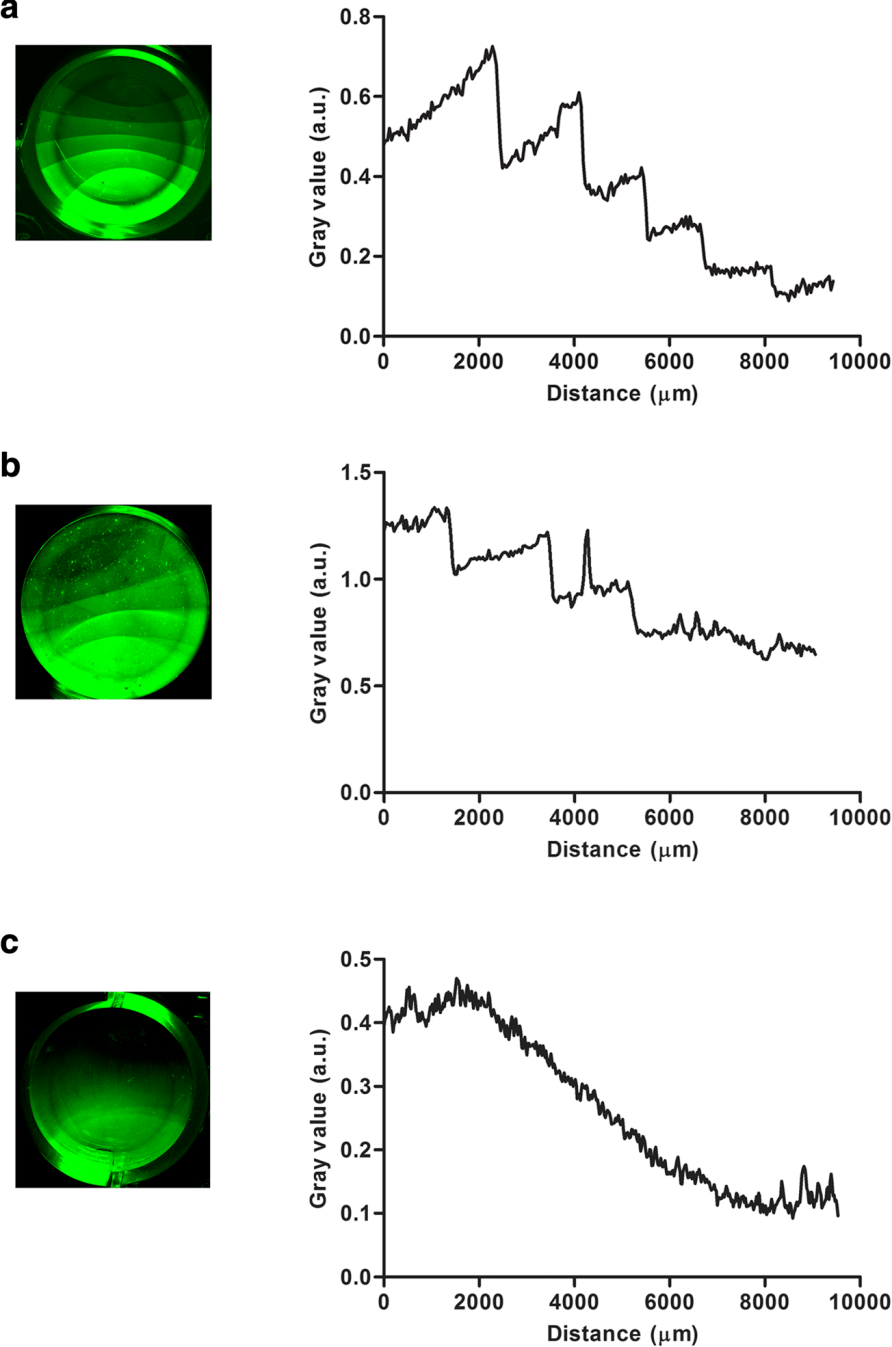
Construction of gradients of heparin and FGF2 in a 24-wells plate (diameter of the well ≈ 1.6 cm). (**a**) A block gradient of heparin created by the stepwise dilutions of 2 μg heparin with 80% (v/v) saturated (NH_4_)_2_SO_4_ solution (see text for details). (**b**) A block gradient of FGF2 created by incubation of a heparin block gradient with 80 ng FGF2 in 0.4% (w/v) BSA in PBS for 1 h. (**c**) A continuous gradient of heparin created by continuously diluting 2 μg heparin with 80% (v/v) saturated (NH_4_)_2_SO_4_ solution using a pump device at a rate of 0.1 ml/h (see text for details). Heparin and FGF2 were immunohistochemically detected using HS4C3V and rabbit anti-fibroblast growth factor respectively and visualized with IgG conjugated IRDye 800CW on a Odyssey CLx imaging system. Using Fiji 1.51n software a gradient profile was plotted by drawing a line from high to low concentrations of heparin. The gray value (a.u.) was determined along the distance of this line. Distances, spanning the gradient, are indicated in μm’s. a.u. = arbitrary units

In order to demonstrate the versatility of the immobilization method, two different types of GAG gradients were created. Since the immobilization of the GAGs is concentration dependent, we prepared gradients of GAGs on surfaces by a dilution strategy. A block gradient of heparin was created by pipetting a concentrated heparin stock solution in 80% (v/v) saturated (NH_4_)_2_SO_4_ at one side of the well in a 24-wells plate followed by a stepwise dilution the heparin stock at the same position with the immobilization solution over time (Fig. 5a). For each step heparin was diluted with a volume of 100 μl of 80% (v/v) saturated (NH_4_)_2_SO_4_ followed by 1-h incubation. The hydrophobicity of the well and surface tension causes the heparin solution to maintain its droplet shape during al dilution steps. This expanding droplet pattern is clearly visible when visualizing the obtained block gradient of heparin with immunohistochemical IRDye staining, with each consecutive “block” with decreasing intensity of the IRDye representing one dilution step. Using the heparin block gradient as a template, a gradient of FGF2 was obtained by incubation with FGF2 in the presence of a low amount of BSA to prevent aspecific binding to the plate (Fig. 5b). The FGF2 gradient followed the pattern of the heparin block gradient. Finally, a continuous gradient of heparin was prepared. A heparin stock solution in 80% (v/v) saturated (NH_4_)_2_SO_4_ was pipetted to one side of a well and continuously diluted with the immobilization solution from the same starting position using a pump system (Fig. 5c).

### Creating patterns of immobilized heparin and CSC

**Fig. 6:**
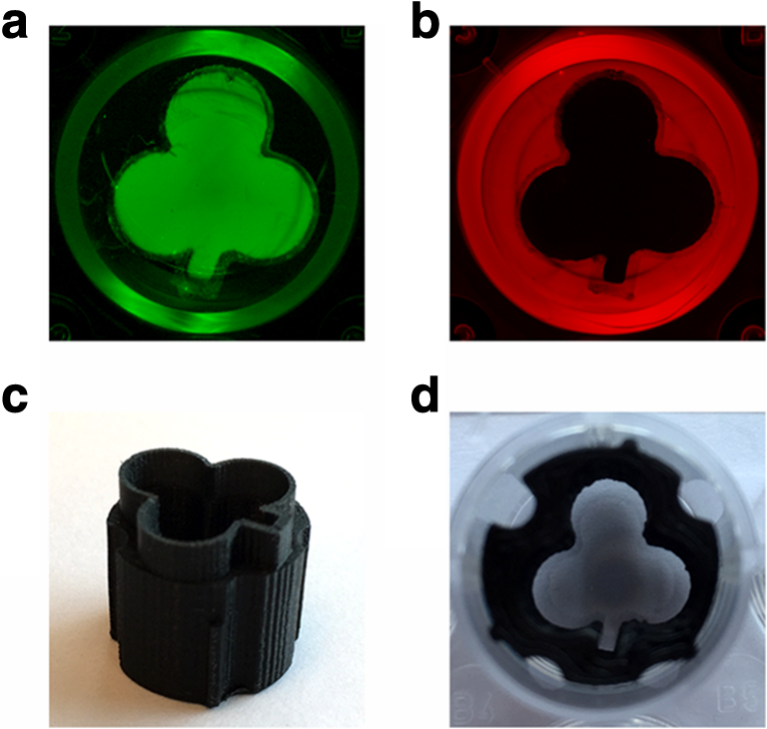
Creation of patterns of immobilized glycosaminoglycans (GAGs) in a 24-wells plate (diameter of the well ≈ 1.6 cm). (**a**) Clover-shaped pattern of heparin. (**b**) Reverse clover-shaped pattern of chondroitin-6-sulfate. (**c**) 3D printed clover-shaped, polylactic acid mold. (**d**) Mold placed at the bottom of a 24-wells plate. Patterns were obtained by pipetting the GAGs (8 μg/well) inside or outside the mold and immobilization with 80% (v/v) saturated (NH_4_)_2_SO_4_ solution. Heparin was immunohistochemically detected using HS4C3V in combination with IgG conjugated IRDye 800CW and CSC using IO3H10 in combination with IgG conjugated with IRDye 680CW followed by visualization on the Odyssey CLx imaging system

Besides gradients, customized patterns of GAGs can be created on surfaces. To illustrate this, a clover-shaped mold was 3D printed, which tightly fitted in a well of a 24-wells plate (Fig. 6c, d). Heparin in 80% (v/v) saturated (NH_4_)_2_SO_4_ was pipetted inside the mold, while CSC in 80% (v/v) saturated (NH_4_)_2_SO_4_ was pipetted in another well on the outside of the mold. In this manner, a clover pattern of heparin and a reverse clover pattern of CSC were obtained as visualized by immunohistochemistry (Fig. 6a, b).

## Discussion

In this study, we demonstrated the efficient and low-cost immobilization of unmodified GAGs onto plastic surfaces using highly concentrated salt solutions. We hypothesize that the applied salt removes the water coat from GAGs and shields their negatively charges, thereby strongly improving hydrophobic interactions of GAGs with surfaces [20]. After washing away the salt, the GAGs are immobilized to the surface as demonstrated immunohistochemically using single chain antibodies.

The efficiency of the GAG immobilization depends on the nature of the salt. Based on their ability to precipitate or solubilize proteins, salts are considered either cosmotropic or chaotropic according to the ordering of the Hofmeister series [27, 28]. It could be hypothesized that the cosmotropic salts of the Hofmeister series are more efficient in immobilizing GAGs as they have the best salting-out and protein-stabilizing characteristics. Indeed, (NH_4_)_2_SO_4_, with both ions at the cosmotropic end of the Hofmeister series, was the most efficient salt. Vlachy et al. proposed a Hofmeister-like ordering of anionic headgroups and their interaction with cations, suggesting that ammonium will form closer ion pairs with sulfate compared with other cations e.g. sodium [29]. This close ion pair formation will lead to less hydration and hence increased hydrophobic interactions. This may explain why (NH_4_)_2_SO_4_ has a higher GAG immobilization efficiency even compared to salts with a higher molarity at saturation, such as LiCl, which would mean a larger number of effective anions and cations to immobilize the GAGs (see Supplementary Table 1).

After immobilization of the GAGs, they were still able to bind the growth factors FGF2 and VEGF, which are both important in wound healing and angiogenesis [30, 31]. Please note that observed differences in growth factor binding amongst the various GAGs may be caused by differences in immobilization efficiency for each GAG. Beside growth factor binding sites, we demonstrated that the cleavage sites of heparin, HS, CSC and DS were available for digestion by GAG lyases. Since the immobilized GAGs remain bioavailable, the described method is suitable to study the GAG-protein interactions underlying biological processes.

Gradients of growth factors are crucial in a variety of biological processes including embryonic development, angiogenesis and inflammatory cell migration [32–34]. We demonstrated that two different types of heparin gradients and various GAG patterns can be created using our immobilization method. The heparin gradient can be used to create gradients of growth factors, as shown for FGF2. Different types of gradients and patterns of GAGs can be used to study the role of growth factor gradients on cellular behavior. For example, Wu et al. demonstrated that the migration rate of vascular smooth muscle cells is dependent on the density of the FGF2 gradient [35].

In conclusion, we provide a straightforward method for the immobilization of unmodified GAGs and the formation of gradients of GAGs and of GAG-binding molecules. The method may be generally applicable to study the biological roles of GAGs.

## Electronic supplementary material

ESM 1 (PDF 241 kb)

## Abbreviations

AP: alkaline phosphatase
BSA: bovine serum albumin
CSC: chondroitin-6-sulfate
DS: dermatan sulfate
ECM: extracellular matrix
GAG: glycosaminoglycan
HS: heparan sulfate
scFv: single chain variable fragment

## References

[1] Bishop JR, Schuksz M, Esko JD (2007). Heparan sulphate proteoglycans fine-tune mammalian physiology. Nat New Biol.

[2] Divya P, Krishnan LK (2009). Glycosaminoglycans restrained in a fibrin matrix improve ECM remodelling by endothelial cells grown for vascular tissue engineering. J. Tissue Eng. Regen. Med..

[3] Gill S, Wight TN, Frevert CW (2010). Proteoglycans: key regulators of pulmonary inflammation and the innate immune response to lung infection. Anat Rec (Hoboken).

[4] Gray E, Mulloy B, Barrowcliffe TW (2008). Heparin and low-molecular-weight heparin. Thromb. Haemost..

[5] Ariga T, Miyatake T, Yu RK (2010). Role of proteoglycans and glycosaminoglycans in the pathogenesis of Alzheimer's disease and related disorders: Amyloidogenesis and therapeutic strategies—a review. J. Neurosci. Res..

[6] Afratis N, Gialeli C, Nikitovic D, Tsegenidis T, Karousou E, Theocharis AD, Pavão MS, Tzanakakis GN, Karamanos NK (2012). Glycosaminoglycans: key players in cancer cell biology and treatment. FEBS J..

[7] Annoni R, Lanças T, Yukimatsu Tanigawa R, de Medeiros Matsushita M, de Morais Fernezlian S, Bruno A, Fernando Ferraz da Silva L, Roughley PJ, Battaglia S, Dolhnikoff M, Hiemstra PS, Sterk PJ, Rabe KF, Mauad T (2012). Extracellular matrix composition in COPD. Eur. Respir. J..

[8] Scharnweber D, Hübner L, Rother S, Hempel U, Anderegg U, Samsonov SA, Pisabarro MT, Hofbauer L, Schnabelrauch M, Franz S, Simon J, Hintze V (2015). Glycosaminoglycan derivatives: promising candidates for the design of functional biomaterials. J Mater Sci Mater Med.

[9] Brouwer KM, Wijnen RM, Reijnen D, Hafmans TG, Daamen WF, van Kuppevelt TH (2013). Heparinized collagen scaffolds with and without growth factors for the repair of diaphragmatic hernia: construction and in vivo evaluation. Organogenesis.

[10] Köwitsch A, Niepel MS, Michanetzis GPA, Missirlis YF, Groth T (2016). Effect of immobilized thiolated glycosaminoglycans on fibronectin adsorption and behavior of fibroblasts. Macromol. Biosci..

[11] Migliorini E, Thakar D, Sadir R, Pleiner T, Baleux F, Lortat-Jacob H, Coche-Guerente L, Richter RP (2014). Well-defined biomimetic surfaces to characterize glycosaminoglycan-mediated interactions on the molecular, supramolecular and cellular levels. Biomaterials.

[12] Pasqui D, Atrei A, Barbucci R (2007). A novel strategy to obtain a hyaluronan monolayer on solid substrates. Biomacromolecules.

[13] Peramo A, Albritton A, Matthews G (2006). Deposition of patterned glycosaminoglycans on silanized glass surfaces. Langmuir.

[14] Wang K, Luo Y (2013). Defined surface immobilization of glycosaminoglycan molecules for probing and modulation of cell–material interactions. Biomacromolecules.

[15] Satoh A, Kojima K, Koyama T, Ogawa H, Matsumoto I (1998). Immobilization of saccharides and peptides on 96-well microtiter plates coated with methyl vinyl ether–maleic anhydride copolymer. Anal. Biochem..

[16] Takada W, Fukushima M, Pothacharoen P, Kongtawelert P, Sugahara K (2013). A sulfated glycosaminoglycan array for molecular interactions between glycosaminoglycans and growth factors or anti-glycosaminoglycan antibodies. Anal. Biochem..

[17] Marson A, Robinson DE, Brookes PN, Mulloy B, Wiles M, Clark SJ, Fielder HL, Collison LJ, Cain SA, Kielty CM, McArthur S, Buttle DJ, Short RD, Whittle JD, Day AJ (2009). Development of a microtiter plate-based glycosaminoglycan array for the investigation of glycosaminoglycan-protein interactions. Glycobiology.

[18] Dickinson, L.E., Gerecht, S.: Micropatterned surfaces to study hyaluronic acid interactions with cancer cells. J Vis Exp. (46), 2413 (2010). 10.3791/2413 10.3791/2413 PMC3159670 21206473

[19] de Paz, J.L., Spillmann, D., Seeberger, P.H.: Microarrays of heparin oligosaccharides obtained by nitrous acid depolymerization of isolated heparin. Chem. Commun. (29), 3116–3118 (2006). 10.1039/B605318A 10.1039/b605318a 16855704

[20] Van Kuppevelt, A.H.M.S.M., Veerkamp, J.H., Blank, T.A.: Method for linking nucleic acids and/or glycosaminoglycans to polar/hydrophilic materials. United States Patent US006933379B2

[21] Van Kuppevelt, A.H.M.S.M., Van de Lest, C.H.A., Veerkamp, J.H.: Method for linking negatively charged macrobiomolecules to plastics, resulting linked compositions and microtitre plates incorporating same. United States Patent US006180769B1

[22] Leenders WPJ, van Hinsbergh VWM, van Genesen ST, Schoenmakers JGG, van Zoelen EJJ, Lubsen NH (1997). Mutants of basic fibroblast growth factor identify different cellular response programs. Growth Factors.

[23] Smetsers TFCM, van de Westerlo EMA, ten Dam GB, Overes IM, Schalkwijk J, van Muijen GNP, van Kuppevelt TH (2004). Human single-chain antibodies reactive with native chondroitin sulfate detect chondroitin sulfate alterations in melanoma and psoriasis. J Invest Dermatol.

[24] van Kuppevelt TH, Dennissen MABA, van Venrooij WJ, Hoet RMA, Veerkamp JH (1998). Generation and application of type-specific anti-heparan sulfate antibodies using phage display technology: further evidence for heparan sulfate heterogeneity in the kidney. J. Biol. Chem..

[25] Dennissen MABA, Jenniskens GJ, Pieffers M, Versteeg EMM, Petitou M, Veerkamp JH, van Kuppevelt TH (2002). Large, tissue-regulated domain diversity of heparan sulfates demonstrated by phage display antibodies. J. Biol. Chem..

[26] ten Dam GB, Yamada S, Kobayashi F, Purushothaman A, van de Westerlo EMA, Bulten J, Malmström A, Sugahara K, Massuger LF, van Kuppevelt TH (2009). Dermatan sulfate domains defined by the novel antibody GD3A12, in normal tissues and ovarian adenocarcinomas. Histochem. Cell Biol..

[27] Hofmeister F (1888). Zur Lehre von der Wirkung der Salze. Arch Exp Pathol Pharmacol.

[28] Kunz W, Henle J, Ninham BW (2004). ‘Zur Lehre von der Wirkung der Salze’ (about the science of the effect of salts): Franz Hofmeister's historical papers. Curr. Opin. Colloid Interface Sci..

[29] Vlachy N, Jagoda-Cwiklik B, Vácha R, Touraud D, Jungwirth P, Kunz W (2009). Hofmeister series and specific interactions of charged headgroups with aqueous ions. Adv. Colloid Interf. Sci..

[30] Hosper NA, Eggink AJ, Roelofs LAJ, Wijnen RMH, van Luyn MJA, Harmsen MC, Geutjes PJ, Daamen WF, van Kuppevelt TH, Tiemessen DM, Oosterwijk E, Crevels JJ, Blokx WAM, Lotgering FK, van den Berg PP, Feitz WFJ, Bank, R.A (2010). Intra-uterine tissue engineering of full-thickness skin defects in a fetal sheep model. Biomaterials.

[31] Nillesen STM, Geutjes PJ, Wismans R, Schalkwijk J, Daamen WF, van Kuppevelt TH (2007). Increased angiogenesis and blood vessel maturation in acellular collagen–heparin scaffolds containing both FGF2 and VEGF. Biomaterials.

[32] Xu P-F, Houssin N, Ferri-Lagneau KF, Thisse B, Thisse C (2014). Construction of a vertebrate embryo from two opposing morphogen gradients. Science.

[33] Halilovic I, Wu J, Alexander M, Lin F (2015). Neutrophil migration under spatially-varying chemoattractant gradient profiles. Biomed. Microdevices.

[34] Guo X, Elliott CG, Li Z, Xu Y, Hamilton DW, Guan J (2012). Creating 3D angiogenic growth factor gradients in fibrous constructs to guide fast angiogenesis. Biomacromolecules.

[35] Wu J, Mao Z, Hong Y, Han L, Gao C (2013). Conjugation of basic fibroblast growth factor on a heparin gradient for regulating the migration of different types of cells. Bioconjug. Chem..

