# Supplementary material for: A versatile salt-based method to immobilize glycosaminoglycans and create growth factor gradients
Source: Glycoconj J. 2019 May 4;36(3):227–36. doi: 10.1007/s10719-019-09872-4 (PMC6548755; doi:10.1007/s10719-019-09872-4)
Supplement: Supplementary file 1 — (PDF 241 kb) [file 10719_2019_9872_MOESM1_ESM.pdf]

A versatile salt-based method to immobilize glycosaminoglycans and create growth factor gradients

Danique J. Hof, Elly M.M. Versteeg, Chris H.A. van de Lest, Willeke F. Daamen, Toin H. van Kuppevelt[1, 2]

Supplementary Table 1 Calculated molarities of 80% (v/v) saturated salt solutions

Salts	Maximum solubility in water (g/L)	80% (v/v) saturated solution (g/L)	Calculated molarity of 80% (v/v) saturated solution (mol/L)
**$(NH_4)_2SO_4$**	764[a]	611	4.63
**$(Na)_2SO_4$**	195[b, c]	156	1.10
**$MgSO_4$**	360[b, c]	288	2.39
**$CaSO_4$**	3.0[a]	2.4	0.022
**NH_4Cl**	395[a]	316	5.91
**KCl**	355[a]	284	3.81
**NaCl**	360[a]	288	4.93
**LiCl**	845[a]	676	15.95
**$MgCl_2$**	543[a, c]	434	4.56
**$CaCl_2$**	745[a, c]	596	5.37
**GuHCl**	215[a, c]	172	1.80

[a] Data derived from Pubchem (National Center for Biotechnology, National Institute of Health USA)
[b] Data derived from the solubility Table of Wikipedia, the Free Encyclopedia
[c] Maximum solubility in water was determined at 20ºC instead of 25ºC

[1] Department of Biochemistry, Radboud Institute for Molecular Life Sciences, Radboud university medical center, PO Box 9101, 6500 HB Nijmegen, The Netherlands
[2] The author to whom correspondence should be addressed: e-mail: Toin.vanKuppevelt@radboudumc.nl
